# Matrix Metalloproteinases 2 and 9 Polymorphism in Patients With Myeloproliferative Diseases: A STROBE-Compliant Observational Study

**DOI:** 10.1097/MD.0000000000000732

**Published:** 2015-04-24

**Authors:** Senem Maral, Muradiye Acar, Ozlem Sahin Balcik, Eyyup Uctepe, Omer Faruk Hatipoglu, Derya Akdeniz, Hatice Uludag Altun, Ali Kosar, Mehmet Gunduz, Esra Gunduz

**Affiliations:** From the Department of Internal Medicine (SM, DA); Division of Hematology (OSB, AK); Department of Medical Genetics (MA, EU, OFH, MG, EG); Department of Medical Microbiology (HUA); and Department of Otolaryngology (MG), Turgut Özal University Faculty of Medicine, Ankara, Turkey.

## Abstract

Chronic myeloproliferative disorders such as polycythemia vera (PV), essential thrombocytosis (ET), and idiopathic myelofibrosis arise from clonal proliferation of neoplastic stem cells in the bone marrow. Matrix metalloproteinases (MMPs) are a family of zinc-dependent endopeptidases that have potential to degrade all types of extracellular matrix (ECM) and also play a role in remodeling of the ECM. It is known that MMPs play a role in bone marrow remodeling.

The primary goal of our study is to explore the relationship between chronic myeloproliferative diseases and some of *MMP* gene polymorphisms. The demonstration of a relationship will help to understand whether these polymorphisms may be a potential early diagnosis marker of the diseases.

Patients were selected from outpatient clinics of Turgut Ozal University Hospital, Ankara, Turkey, between December 2010 and May 2011. Twenty-eight patients that previously diagnosed and followed-up with PV, 17 with secondary polycythemia (SP), and 12 with ET were enrolled in the study, along with a control group of 22 healthy people.

DNA was isolated from peripheral blood. Using polymerase chain reaction–restriction fragment length polymorphism method, *MMP2* and *MMP9* gene polymorphisms were analyzed with agarose gel electrophoresis. There was a statistically significant difference between the study groups and the control group in terms of Gln279Arg polymorphisms rates of MMP9. The highest MMP9 Gln279Arg polymorphism rate was observed in the ET group. But nobody from the control group had polymorphic MMP9. There was no statistically significant difference between the groups in terms of MMP2-735 C > T polymorphism rates.

In conclusion, *MMP9* gene Gln279Arg polymorphism was associated with ET, SP, and PV diseases. Hence, we believe that these gene polymorphisms may play a role in the mechanism of bone marrow fibrosis and may be a factor that increases the risk of thrombosis. Illumination of the molecular basis of the relationship between MMP-thrombosis and MMP-fibrosis provides a better understanding of the pathophysiology of PV and ET diseases and will allow new approaches to diagnosis and treatment.

## INTRODUCTION

Chronic myeloproliferative disorders (MPDs) are characterized by progressive remodeling of bone marrow stroma as evidenced by increased deposition of extracellular matrix (ECM) proteins, neoangiogenesis, and displacement of normal hematopoietic cells by fibrotic tissue. Polycythemia vera (PV), essential thrombocytosis (ET), and idiopathic myelofibrosis are disorders caused by clonal proliferation of abnormal neoplastic stem cells in bone marrow. These diseases usually appear in middle-aged people and may transform to acute leukemia.

Matrix metalloproteinases (MMPs) are a family of zinc and calcium-dependent endopeptidases that are known to be integral for not only the remodeling of the ECM but also its degradation. Regulations of these molecules are arranged by specific tissue inhibitors called tissue inhibitory metalloproteinases (TIMPs) containing α2 macroglobulin.^1–3^ TIMPs inhibit matrix degradation processes. Both TIMPs and MMPs can be secreted from stromal and tumoral cells. According to substrate specificity, MMPs are divided into 4 groups: collagenases, gelatinases, stromelysins, and membrane-type metalloproteinases. Gelatinases, which are called MMP2 and MMP9, digest denatured collagen and gelatine structures. The gelatinases are the only MMPs that contain 3 contiguous fibronectin type II homology units, collectively known as the fibronectin-like domain, that are inserted into their catalytic domains. Several reports have indicated that this domain plays a role in the various proteolytic activities of these enzymes. It has been shown that the presence of this domain greatly increases their gelatinolytic and elastinolytic activities.^4–6^ Unlike MMP9, MMP2 is highly potent in the degradation of collagen types I, II, and III.^7,8^ However, both MMP types break down type IV collagen that is the major structural component of the basement membrane.^9^

MMP enzyme is encoded by *MMP2* gene with 13 exons, located on 16q12.2.^10^ Many tissues express the MMP2 enzyme such as oral tissues (gingival odontoblasts and osteoblasts) and hematopoietic cells (erythroblasts, myeloid cells, and megakaryocytes).^9,11^ Functionally, this enzyme has been shown to play a role in endometrial menstrual breakdown, regulation of vascularization, the inflammatory response, and osteogenesis but they are also vital in pathological roles such as tumor invasion and metastasis.^12,13^ Enhanced invasive capacity of tumor cells occurs when soluble MMP2 bind to the surface of these cells (in vitro and in vivo) by interaction with the integrin receptor avb4.^14^

There are 2 variant isoforms of this enzyme and are due to 2 functional promoter single-nucleotide polymorphisms (SNPs) in which cytosine is replaced by thymine (rs243865: −1306 C > T, and rs2285053: −735 C > T).^11^ rs2285053 (−735 C > T) have been previously reported to affect MMP2 transcription in vitro; both C to T transitions result in reduced expression due to the ablation of specificity protein transcription factor-binding sites.^15,16^In addition to normal processes, MMPs have roles in some pathological processes, such as tumor invasion and metastasis.^13^ Soluble MMP2 binds to the surface of invasive cells in vitro and in vivo by interaction with the integrin receptor avb3, which enhances the invasive capacities of these cells.^14^

MMP9, also known as 92 kDa gelatinase and type V collagenase, could degrade type IV collagen, which is an important component of the ECM.^17^ It is encoded by *MMP9* gene with 13 exons, locating at 20q11.2-q13.1,^10^ and it could be produced by normal mononuclear cells, granulocytes, smooth muscle cells, vascular endothelial cells, and multiple other types of cells. SNPs in MMP9 might cause a change in function of MMP9 and thus affect ECM remodeling.

**FIGURE 1:**
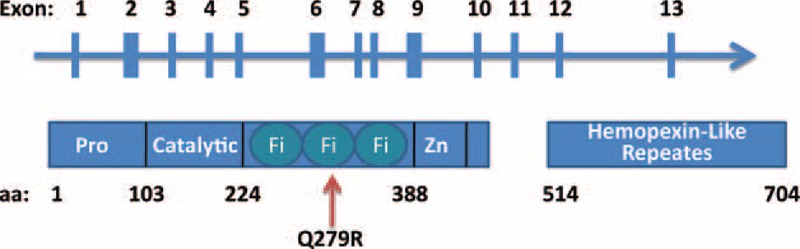
Presentation of *MMP9* gene and Q279R polymorphism. MMP = matrix metalloproteinase.

SNP in MMP rs17576 (c836G>A, p.279 Q > R) leads to a substitution of an uncharged amino acid (glutamine) by a positively charged amino acid (arginine) that resides within the substrate-binding portion of the highly conserved gelatinase-specific fibronectin type II domain (FN2) (Fig. 1)^4,18,19^ and located in the substrate-binding domain of this enzyme.^4,19^ FN2 is 1 of 3 types of internal repeats that combine to form larger domains within fibronectin. Fibronectin, a plasma protein that binds cell surfaces and various compounds including collagen, fibrin, heparin, DNA, and actin, usually exists as a dimer in plasma and as an insoluble multimer in extracellular matrices. The FN2 of MMP9 was found to have affinity for denatured collagen.^20^ Using FASTSNP software to predict functional effects of the amino acid change from glutamine to arginine induced by rs2664538, this gene variation seems highly likely to change protein structure and exonic splicing.^21^ In our study, we aimed to evaluate MMP2 and MMP9 polymorphism and disease associations in patients with PV, ET, and secondary polycythemia (SP).

## METHODS

Patients were selected from outpatient clinics of Turgut Ozal University Faculty of Medicine, Ankara, Turkey, between December 2010 and May 2011. Twenty-eight patients with PV, 17 patients with SP, and 12 patients with ET diagnosed and followed by hematology outpatient clinics between 25 and 81 of ages and 22 age-matched control were included (male/female: 27/52, for all groups). Complete blood count test was performed for all patients. JAK2 mutations were studied to differentiate MPDs from reactive hematopoietic diseases. Then, MMP2 and MMP9 polymorphism were studied for each patient. All samples were collected after acquisition of informed consent from each patient and approval of the study by the Institutional Human Ethics Committee of Turgut Özal University.

### DNA Isolation

DNA was isolated from peripheral blood via invitrogen PureLink Genomic DNA Kit (Invitrogen, USA) for purification of genomic DNA (K1820-02) kit. Obtained DNAwas stored at −20 °C until it was used in polymerase chain reaction (PCR). Using PCR–restriction fragment length polymorphism (PCR-RFLP) method, *MMP2* and *MMP9* gene polymorphisms were analyzed for study and control groups.

### DNA Amplification

We analyzed *MMP2* gene −735 C > T (rs2285053) with primers F: 5′-GGATTCTTGGCTTGGCGCAGGA-3′ and R: 5′-GGGGGCTGGGTAAAATGAGGCTG-3′ for rs2285053 at Techne TC-5000 model PCR device. The PCR was carried out in a 25 μL reaction volume containing 2.5 μL 10X buffer, 1.3 μL MgCl_2_ (25 mM), 0.5 μL Deoxynucleotide triphosphates (dNTPs), 0.5 μL of each primer (20 pmol), 17.4 μL H_2_O, and 0.3 μL of Taq DNA polymerase (Thermo scientific Cat no: #EPO402) that were distributed to each reaction tube and then 2 μL DNA sample was added to each sample. The PCR conditions were as follows: initial denaturation at 95°C for 5 minutes, 36 cycles of denaturation at 95°C for 30 seconds, annealing at 67°C for 30 seconds, extension at 72°C for 1 minute, and a final extension at 72°C for 5 minutes.

We also analyzed *MMP9* gene Gln279Arg (rs17576 G > A) polymorphisms with primers F: 5′-AGACCATCCATGGGTCAAAG-3′ and R: 5′-GATTGGCCTTGGAAGATGAA-3′. The PCR was carried out in a 25 μL reaction volume containing 2.5 μL 10X buffer, 2.5 μL MgCl_2_ (25 mM), 0.5 μL dNTPs, 0.3 μL of each primer (20 pmol), 16.8 μL H_2_O, and 0.1 μL of Taq DNA polymerase (Thermo scientific Cat no: #EPO402) that were distributed to each reaction tube and then 2 μL DNA sample was added to each sample. The PCR conditions were as follows: initial denaturation at 95°C for 5 minutes, 30 cycles of denaturation at 95°C for 30 seconds, annealing at 60°C for 30 seconds, extension at 72°C for 1 minute, and a final extension at 72°C for 5 minutes.

### Agarose Gel Electrophoresis

**FIGURE 2:**
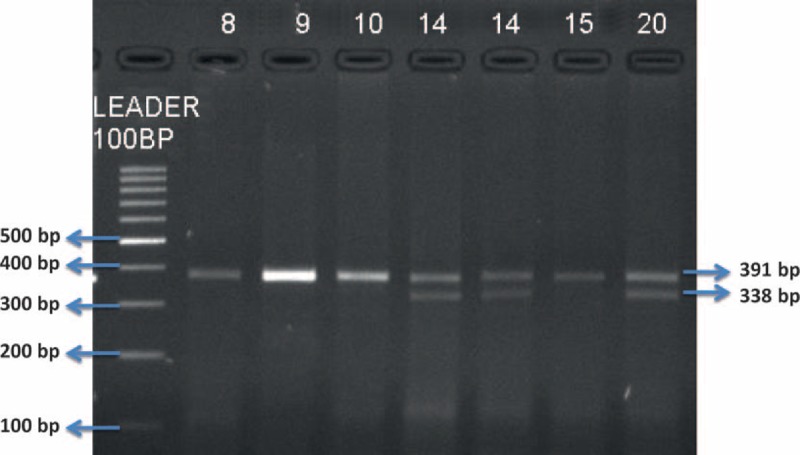
MMP2 −735 CT polymorphism agarose gel electrophoresis image after RFLP. MMP = matrix metalloproteinase, RFLP = restriction fragment length polymorphism.

**FIGURE 3:**
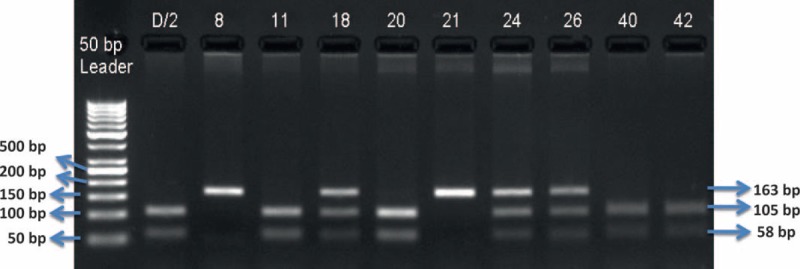
MMP9 Gln279Arg gene polymorphism agarose gel electrophoresis image after RFLP. MMP = matrix metalloproteinase, RFLP = restriction fragment length polymorphism.

Three percent agarose gel was prepared for running the samples. Agarose gel electrophoresis was first performed to control amplification of PCR products and then to detect alleles after RFLP (Figs. 2 and 3).

### Enzymatic Digestion of DNA

Targeted SNP were investigated by restriction enzymes that recognize this region and the alleles were identified.

#### Enzymatic Digestion for MMP2 −735 C > T Polymorphism

Amplified genome segment was 391 bp lengths and was cut into 338 and 53 bp pieces by Hinf I (NEB R0155S) enzyme. Enzyme cutting point was T nucleotide. Length of PCR product after RFLP was 391 bp for CC genotype, 391, 338, and 53 bp for CT genotype, and 338 and 53 bp for TT genotype. Also, these results were controlled with Sanger sequencing.

#### Enzymatic Digestion for *MMP9* Gene Gln279Arg Polymorphism

Amplified genome segment was 163 bp length and was cut into 105 and 58 bp pieces by SmaI (NEB R0141S) enzyme. Enzyme cutting point was G nucleotide. Length of PCR product after RFLP was 163 bp for AA genotype, 163, 105, and 58 bp for AG genotype, and 105 and 58 bp for GG genotype. Also, these results were controlled with Sanger sequencing.

### Statistical Analysis

All analyses were performed by using statistical program (SPSS) for Windows, version 15.0 packed programs (SPSS Inc., Chicago, IL). Parameters were not normally distributed. Significance of difference between the groups in terms of average variance (one-way analysis of variance) and median values was determined by Kruskal–Wallis test. Data were announced as mean value ± standard deviation. Cross table was created to investigate the relationship between study groups and categorical variables (gender, MMP2, and MMP9), and differences between groups were evaluated with χ^2^ test. *P* values of ≤0.05 were interpreted as statistically significant.

## RESULTS

In our study, there were 4 groups: PV (n = 28), SP (n = 17), ET (n = 12), and control group (n = 22); 65.8% of all patients were men. Mean age of PS group was 58.5 and of ET group was 52 years. There was no significant difference between ages of all groups. Demographic features of all patients are listed in Table 1.

**TABLE 1:**
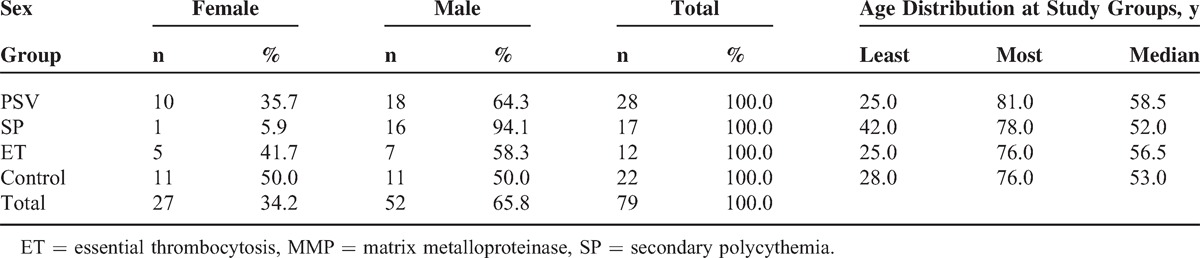
Demographic Features of Patients

There was a statistically significant difference between the patient groups and the control groups in terms of MMP9 Gln279Arg A > G polymorphisms (*P* < 0.05). Table 2 displays the distribution of genotypes and frequency of alleles of the MMP2 −735 C > T and MMP9 Gln279Arg polymorphisms in patients with PV, SP, ET, and controls.

**TABLE 2:**
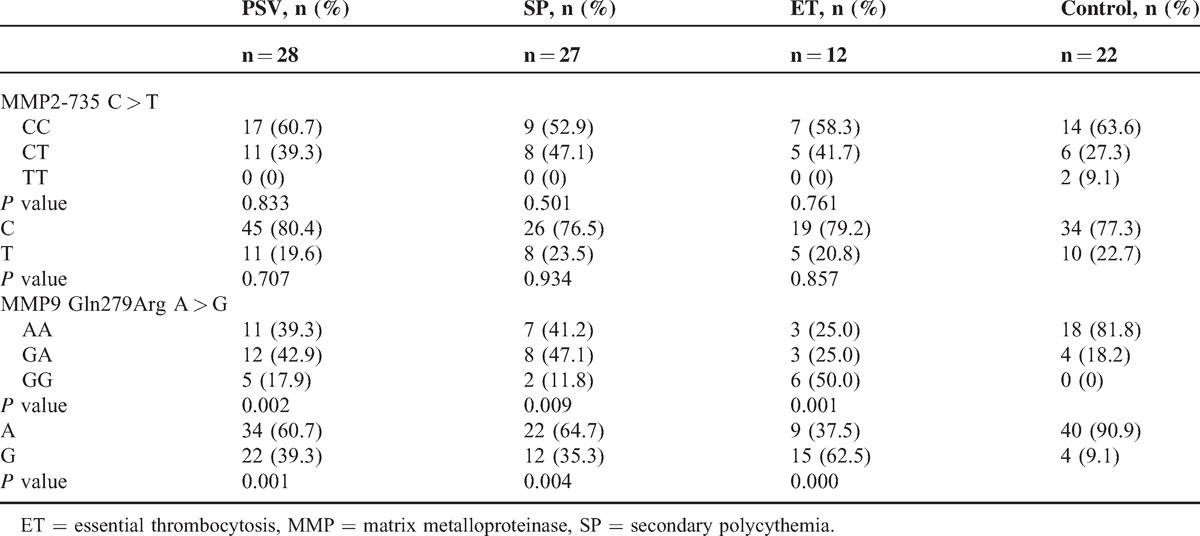
Allele and Genotype Frequencies of MMP2 and MMP9 Gene Polymorphism in Study Groups

**FIGURE 4:**
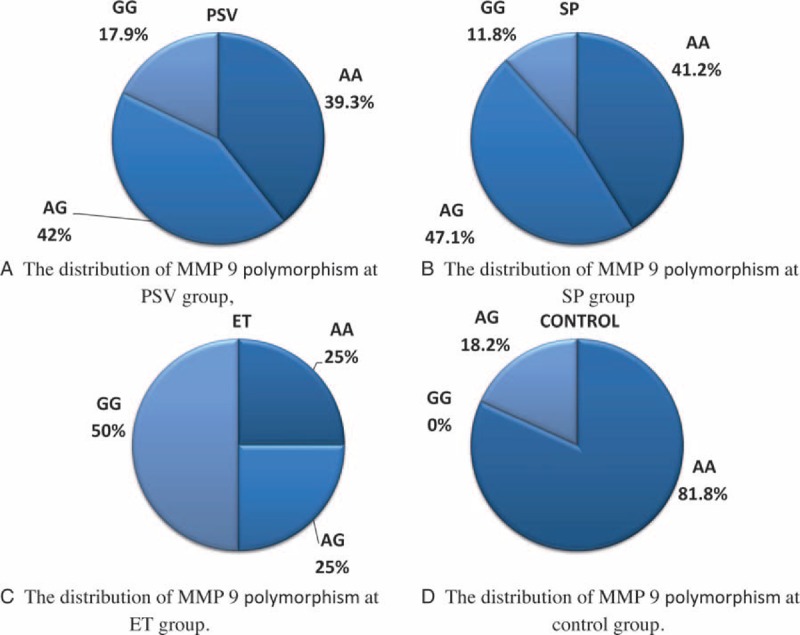
Distributions of MMP9 polymorphism at study groups. (A) At PV. (B) At SP. (C) At ET. (D) At control. ET = essential thrombocytosis, MMP = matrix metalloproteinase, SP = secondary polycythemia.

The frequency of MMP9 Gln279Arg A > G polymorphism genotypes were AA 39.3% (n = 11), AG 42.9% (n = 12), GG 17.9% (n = 5), and *P* = 0.002 in PV patients (Fig. 4). Also MMP9 genotypes frequencies in SP patients were AA 41.2% (n = 7), AG 47.1% (n = 8), GG 11.8% (n = 2), and *P* = 0.009. Finally MMP9 genotypes frequencies in ET patients were AA 25% (n = 3), AG 25% (n = 3), GG 50% (n = 6), and *P* = 0.001. Also MMP9 genotypes frequencies in control groups were AA 81.8% (n = 18), AG 18.2% (n = 4), and GG 0% (n = 0). The allele frequency of MMP9 Arg279Gln A > G in PV was A 60.7% (n = 34), G 39.3% (n = 22), and *P* = 0.001; in SP was A 64.7% (n = 22), G 35.3% (n = 12), and *P* = 0.004; in ET was A 37.5% (n = 9), G 62.5% (n = 15), and *P* < 0.001; and in control group was A 90.9% (n = 40) and G 9.1% (n = 4).

**FIGURE 5:**
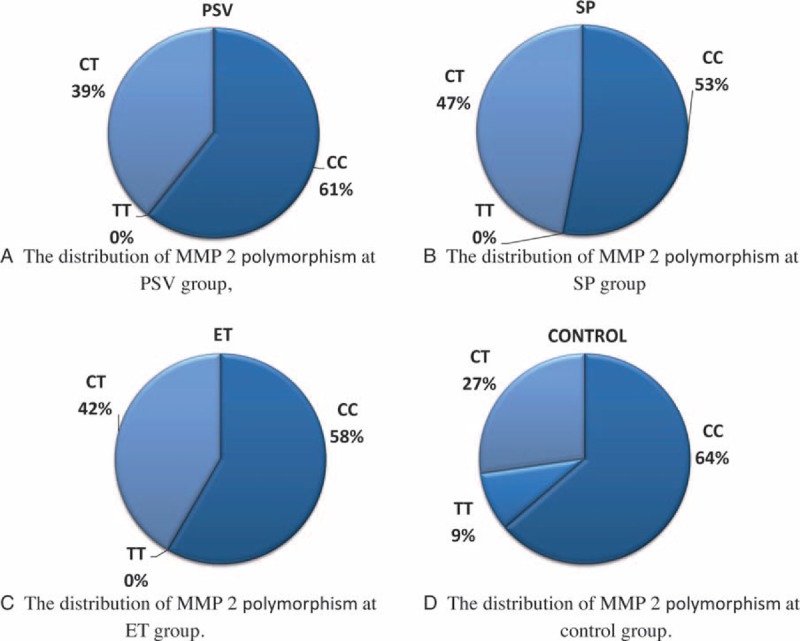
Distributions of MMP2 polymorphism at study groups. (A) At PV. (B) At SP. (C) At ET. (D) At control. ET = essential thrombocytosis, MMP = matrix metalloproteinase, SP = secondary polycythemia

We did not find any significant difference in genotype or allele frequencies in MMP2 −735 C > T polymorphisms in patients with PV, SP, ET, and controls (*P* > 0.05) (Fig. 5). These results are also demonstrated in Table 2.

## DISCUSSION

ECM contains structural proteins such as elastin and collagen. Remodeling of ECM requires proteases. MMPs are investigated for their remodeling effects on embryogenesis, angiogenesis, tumor invasion, metastasis, and atherosclerotic processes.^22,23^ MMP2 (gelatinases A) polymorphism was identified as increased in murine tumors in 1981 by Liotta et al.^24^ This molecule is responsible from destruction of gelatine, fibronectin, and collagen types IV, V, VII, and X. We have studied the *MMP2* gene −735 C > T polymorphism in PV, SP, and ET. We found that MMP2 polymorphism ratio was 39.3% in PV, 47.1% in SP, and 27.3% in the control group. There was no statistically significant difference between the groups. There were only 2 patients carrying TT genotype for *MMP2* gene −735 C > T polymorphism in the control group.

MMP2 −735 C > T polymorphism affects MMP2 expression or activity and have been associated with cardiovascular diseases^16,25,26^ and obesity.^27,28^ In another study, it was shown that blood pressure is associated with circulating MMP2 concentrations, and that the CT genotype and the T allele for the C −735T polymorphism are less common in obesity. However, the haplotypes distribution did not show significant differences between control and obese (*P* > 0.05).^29^ Also polymorphisms in MMP2 (−735 C > T) and MMP9 (−1562 C > T) were associated with elevated risk of endometriosis.^30^

MMP2 and MMP9 are implicated in the invasive phenotype of various malignant neoplasias, such as tumors of the colon, breast, ovary, kidney, or skin.^31–37^ The variant MMP2 genotype (−1306 CT or TT) was correlated with a substantially reduced risk of breast cancer.^38^ Expression of MMP2, 7, and 11 was determined greater in pancreatic carcinoma than in normal pancreas (*P* < 0.01).^39^

A study suggests that genetic variations in the MMP2 (rs2285053) might be potential predictors of distant metastasis-free survival after curative surgery in patients with colorectal cancer.^40^

Another study suggested that genetic variants in *MMP2* (rs2285053), 7, 9, and *TIMP2* genes are associated with higher susceptibility of gallbladder cancer.^41^ Functional polymorphisms in the promoter regions of MMP2 (−735 C > T) and MMP3 are not associated with melanoma progression.^42^ Zhou et al^43^ suggested that the genetic polymorphisms or haplotype in the MMP2 promoter (rs243865, −1306 and rs2285053, −735) might play a role in mediating the susceptibility to nasopharyngeal carcinoma in Chinese populations. For MMP2, a case–control study showed that patients with esophageal squamous cell carcinoma carrying the −1306 CC or −735 CC genotypes had an increased risk of developing cancer.^15^

MMP9 (gelatinases B) Gln279Arg gene polymorphism was found in myeloid cells and megacaryocytic cell lines.^11^ It is secreted from keratinocyte, monocyte, alveolar macrophages, polymorphonuclear leukocytes, and malignant cells. Studies in transgenic mice show that MMP has a role in neoplastic differentiation and neovascularization.^44^ The process of angiogenesis has been correlated with increasing MMP9 levels.^45^ MMP9 has a role in both inflammatory and anti-inflammatory pathways depending on the surrounding cytokines.^46^ It is also associated with increased cell survival of lymphocytes in chronic lymphocytic leukemia.^47^ The MMP9 Gln279Arg polymorphism lies in the substrate-binding region and decreases binding affinity of type IV collagen to MMP9.^4^ MMP9 279Gln > Gln genotypes was found to be associated with increased risk of diabetic nephropathy suggesting that decreased affinity of MMP9 to type IV collagen may lead to decreased degradation with subsequent accumulation of ECM proteins and thereby contributing to renal damage.^48^ This SNP has previously been reported to associate with metastasis of lung cancer^49^ and trachoma.^50^

It is demonstrated that mononuclear cells derived from bone marrow continuously secrete MMP9 and TIMP1. However, leukemic blast cells produce MMP2 that may serve as a marker for dissemination in myeloproliferative malignancies.^51^ Recently, leukemic blast cells purified from the peripheral blood of patients with Acute myeloid leukemia have been demonstrated to regularly release MMP2 and MMP9.^52^ Some studies show the involvement of MMPs and TIMPs in growth and progression of lymphoid neoplasias.^53–55^

In the search for potential markers of the bone marrow remodeling process seen in myelofibrosis patients, 1 group examined the MMP and TIMP levels in these patients.^56^ No statistical significant difference between TIMP2 and MMP2 levels was determined between patients and controls. Median MMP9 concentration was significantly higher among PV patients compared with controls (*P* = 0.0015). Also the ratio of total TIMP1/MMP9 was significantly higher in patients with Myelofibrosis compared with controls (*P* = 0.0004). These findings indicate that an impaired TIMP1/MMP9 ratio may reflect an imbalance of the extracellular homeostasis toward an increased matrix deposition enhancing fibrosis.

In our study, we have found the highest MMP9 Gln279Arg polymorphism rate in the ET group. No patient from the control group has this polymorphism. Our results were compatible with the literature.^56^ It is shown that there was a significant correlation between MMP9 values and platelet count among patients suggesting that plasma MMP9 reflects platelet mass as well as being a marker for circulating granulocytes. Because thrombotic events are increased in patients with ET, there may be some relation between MMP9 polymorphism and increased risk of thrombosis in these patients groups. More studies are needed in this area.

There are some studies that identify the risk between thrombosis and MMP9 levels.^57,58^ We also found positive correlation between the thrombotic event history, JAK2 mutation, and MMP9 polymorphism in the ET group (*P* = 0.006 and 0.02). But there was not positive correlation between thrombocyte count and MMP9 polymorphism. This discrepancy may be related to disease treatment or variation in platelet function rather than platelet number.

As conclusion, we have found increased polymorphism of MMP9 in PV, SP, and ET groups. This parameter may be related to risk of thrombosis in these groups. But more studies are needed on this subject.
